# The ratio of single- to double-strand DNA breaks and their absolute values determine cell death pathway

**DOI:** 10.1054/bjoc.2001.1786

**Published:** 2001-05

**Authors:** O Tounekti, A Kenani, N Foray, S Orlowski, L M Mir

**Affiliations:** L.P.P.M.B., UMR 8532 CNRS, Institut Gustave Roussy, PR II, 39, rue Camille Desmoulins, F-94805 Villejuif Cédex, France; Institut National des Sciences Appliquées et de Technologie, rue M. Ali Akid, B.P. 676, 1080 Tunis, Tunisia; Laboratoire de Biochimie, Faculté de Médecine de Monastir, 5019, Monastir, Tunisia; Institut Curie – U350 INSERM, Centre Universitaire, F-91405 Orsay, France; BPM/DBCM, CEA and URA 2096 CNRS, Centre d'Etudes de Saclay, F-91191 Gif/Yvette Cédex, France

**Keywords:** bleomycin, deglyco-bleomycin-A _2_, apoptosis, mitotic cell death, electroporation, electrochemotherapy

## Abstract

Bleomycin is a cytotoxic antibiotic that generates DNA double-strand breaks (DSB) and DNA single-strand breaks (SSB). It is possible to introduce known quantities of bleomycin molecules into cells. Low amounts kill the cells by a slow process termed mitotic cell death, while high amounts produce a fast process that has been termed pseudoapoptosis. We previously showed that these types of cell death are a direct consequence of the DSB generated by bleomycin. Here, we use deglyco-bleomycin, a bleomycin derivative lacking the carbohydrate moiety. Although this molecule performs the same nucleophilic attacks on DNA as bleomycin, we show that deglyco-bleomycin is at least 100 times less toxic to Chinese hamster fibroblasts than bleomycin. In fact, deglyco-bleomycin treatment results in apoptosis induction. In contrast, however, deglyco-bleomycin was found to generate almost exclusively SSB. Our results suggest that more than 150 000 SSB per cell are required to trigger apoptosis in Chinese hamster fibroblasts and that SSB are 300 times less toxic than DSB. Taken together with previous studies on bleomycin, our data demonstrates that cells can die by apoptosis, mitotic cell death, or pseudoapoptosis, depending on the number of DNA breaks and on the ratio of SSB to DSB. © 2001 Cancer Research Campaign http://www.bjcancer.com


*British Journal of Cancer* (2001) **84**(9), 1272–1279
© 2001 Cancer Research Campaign
doi: 10.1054/ bjoc.2001.1786, available online at http://www.idealibrary.com on IDEAL®

http://www.bjcancer.com

### The ratio of single- to double-strand DNA breaks and their absolute values determine cell death pathway

**O Tounekti[1,2], A Kenani[1,3], N Foray[4], S Orlowski[5] and LM Mir[1]**

[1]L.P.P.M.B., UMR 8532 CNRS, Institut Gustave Roussy, PR II, 39, rue Camille Desmoulins, F-94805 Villejuif Cédex, France; [2]Institut National des Sciences Appliquées et de Technologie, rue M. Ali Akid, B.P. 676, 1080 Tunis, Tunisia; [3]Laboratoire de Biochimie, Faculté de Médecine de Monastir, 5019, Monastir, Tunisia; [4]Institut Curie – U350 INSERM, Centre Universitaire, F-91405 Orsay, France; [5]SBPM/DBCM, CEA and URA 2096 CNRS, Centre d'Etudes de Saclay, F-91191 Gif/Yvette Cédex, France

**Summary** Bleomycin is a cytotoxic antibiotic that generates DNA double-strand breaks (DSB) and DNA single-strand breaks (SSB). It is possible to introduce known quantities of bleomycin molecules into cells. Low amounts kill the cells by a slow process termed mitotic cell death, while high amounts produce a fast process that has been termed pseudoapoptosis. We previously showed that these types of cell death are a direct consequence of the DSB generated by bleomycin. Here, we use deglyco-bleomycin, a bleomycin derivative lacking the carbohydrate moiety. Although this molecule performs the same nucleophilic attacks on DNA as bleomycin, we show that deglyco-bleomycin is at least 100 times less toxic to Chinese hamster fibroblasts than bleomycin. In fact, deglyco-bleomycin treatment results in apoptosis induction. In contrast, however, deglyco-bleomycin was found to generate almost exclusively SSB. Our results suggest that more than 150 000 SSB per cell are required to trigger apoptosis in Chinese hamster fibroblasts and that SSB are 300 times less toxic than DSB. Taken together with previous studies on bleomycin, our data demonstrates that cells can die by apoptosis, mitotic cell death, or pseudoapoptosis, depending on the number of DNA breaks and on the ratio of SSB to DSB. © 2001 Cancer Research Campaign http://www.bjcancer.com

**Keywords**: bleomycin; deglyco-bleomycin-$A_2$; apoptosis; mitotic cell death; electroporation; electrochemotherapy

Cell death pathways induced by DNA damage include apoptosis, an active process of cell suicide, as well as mitotic cell death, which results from the passage through mitosis of cells containing unrepaired DNA breaks (Cohen-Jonathan et al, 1999). Ionizing radiation, or chemicals, may produce many types of DNA damage including single-strand breaks (SSB), double-strand breaks (DSB), base damage, and DNA–DNA or DNA–protein crosslinks (Cole et al, 1980). A correlation between cell responses and the various forms of DNA damage has not yet been fully described (Cohen-Jonathan et al, 1999). Cole et al (1975) have presented evidence that repairable SSB probably do not result in cell death, but DSB are considered to be severe lethal events (Goodhead, 1989, Tounekti et al, 1993). Nonetheless, the exact role of DNA breaks in the triggering of cell death is far from being well established.

Bleomycin (BLM) is a radiomimetic anticancer drug (in fact a mixture of 11 compounds, the most abundant being bleomycin-$A_2$ (BLM-$A_2$)) that produces SSB and DSB in a catalytic way. On average, each BLM molecule generates 8 to 10 DNA breaks (Povirk et al, 1989); it is admitted that for every 6 SSB, 1 DSB is created (Greenway and Beerman, 1986; Cullinan et al, 1991). We previously demonstrated in cultured cells that moderate quantities of BLM-generated DSB (between 500 and 50 000 DSB per cell) induce a slow cell death reminiscent of the mitotic cell death caused by ionizing radiations. However, larger numbers of DSB (larger than $5 \times 10^5$, generated by the internalization of more than $3 \times 10^5$ BLM molecules per cell), provoke pseudoapoptosis, that is, a fast cell death process that displays the morphological and biochemical characteristics of apoptosis, directly caused by bleomycin rather than by the relevant endonuclease (Tounekti et al, 1993). The internalization of large amounts of BLM is only achievable after transient and reversible permeabilization of cells, such as is obtained by cell electropermeabilization (EPN) (Orlowski and Mir, 1993). BLM uptake is restricted by the fact that BLM molecules do not diffuse through the plasma membrane (Orlowski et al, 1988; Poddevin et al, 1991; Mir et al, 1996). Cell EPN is an advantageous procedure by which the average number of BLM molecules internalized by the cells may be controlled since this number is proportional to the external BLM concentration at the time of EPN. Furthermore, cell EPN allows the synchronization of the BLM effects just at the time the electric pulses are delivered. The introduction of BLM by this method provides a tool to study the cellular consequences of known amounts of SSB and DSB generated at a given time.

Deglyco-bleomycin-$A_2$ (deglyco-BLM-$A_2$) is a BLM-$A_2$ derivative lacking the carbohydrate moiety (Oppenheimer et al, 1982). It has been shown recently that, like all BLMs, deglyco-BLM-$A_2$ has retained the ability to preferentially recognize the GC sequences on DNA and to produce breaks at this position (Bailly et al, 1995). Thus, deglyco-BLM-$A_2$ and BLM-$A_2$ share an identical mechanism of action at the DNA level. In this report though, we show that, contrary to BLM, deglyco-BLM-$A_2$ induces apoptosis in DC-3F cells and generates essentially SSB. Results obtained in Chinese hamster fibroblasts strongly suggest that the type of cell death depends on both the relative and the absolute quantities of SSB and DSB generated per cell.

*Received 1 September 2000*
*Revised 8 February 2001*
*Accepted 12 February 2001*

*Correspondence to:* LM Mir

### MATERIALS AND METHODS

### Cells and chemicals

Chinese hamster DC-3F fibroblasts were maintained in the previously described culture conditions (Orlowski et al, 1988). Cell culture materials were obtained from Gibco Laboratories (Cergy-Pontoise, France). Lyophilized BLM (Lab. Roger Bellon, Neuilly, France) was dissolved in NaCl 0.9% at pH 7 and stored at –20˚C. BLM-$A_2$ was kindly provided by Nippon Kayaku (Tokyo, Japan). Deglyco-BLM-$A_2$ was prepared by BLM-$A_2$ solvolysis using fluorhydric acid (Kenani et al, 1988b). Deglyco-BLM purity (i.e. the complete deglycosylation of the batch of BLM) was checked by high-pressure liquid chromatography and by ion spray/mass spectrometry, according to Kenani et al (1988). DNase-free RNase and proteinase K were purchased from Boehringer Mannheim (Meylan, France) and all other products from Sigma Chemical Co (La Verpillière, France) except when otherwise stated.

### Cell electropermeabilization procedures

Cell electropermeabilization was performed using a square wave pulse generator (PS-15 electropulsator, Jouan, Saint-Herblain, France). After harvest by trypsinization of exponentially growing cells and inactivation of trypsin by complete medium, cells were washed 3 times in 0.5 mM $Ca^{2+}$-supplemented serum-free S-MEM (Gibco Lab). Cells were then resuspended in the same ice-cooled medium at $2.2 \times 10^7$ cells $ml^{-1}$. Aliquots of 67.5 µl of the cell suspension were mixed with 7.5 µl of 10-fold concentrated drug solutions. 50 µl of the mixture were immediately deposited between two electrodes (2 mm apart) and subjected to the electric treatment (8 pulses of 100 µs and 1250 V $cm^{-1}$ delivered at a frequency of 1 Hz). Then, cells were kept for 5 min at 24˚C, diluted in complete culture medium and seeded or treated as described below.

### Cytotoxicity determinations and cell staining with trypan blue

After EPN, cells were seeded in triplicate (500 cells per cell culture dish 60 mm in diameter) and cultured for 5 days at 37˚C for cloning efficiency assay. Colonies were counted and values obtained were expressed as the percentage of the colonies obtained with control cells treated in the absence of drug. The absolute cloning efficiency of controls was 60 to 70%. To determine cell morphology after the treatment, cells were trypsinized, harvested, stained by the addition of an equal volume of trypan blue solution (0.08% trypan blue and 0.005% p-hydroxybenzoic acid methyl, sodium salt in phosphate buffered saline, PBS). Cells were then observed in a haemocytometer under a phase-contrast light microscope using 160x magnification.

### Treatments with apoptosis inhibitors

One hour after cell EPN in the presence of deglyco-BLM-$A_2$, aurintricarboxylic acid (0.5–100 µM) or $ZnCl_2$ (0.1–5 mM) or $CoCl_2$ (0.1–5 mM) were added to the culture medium and left until cells were collected. After various incubation times at 37˚C, cells were processed to analyse DNA fragmentation as described below.

### DNA damage analysis by alkaline elution

Cells were labelled for 24 h with [2-$^{14}$C]thymidine (0.02 µCi $ml^{-1}$). They were then electropermeabilized in the absence or presence of 0.1, 1 or 10 µM deglyco-BLM-$A_2$ and incubated for 20 min at room temperature. DC-3F cells labelled for 24 h with [$^3$H]thymidine (0.2 µCi $ml^{-1}$) were irradiated (2000 rad) on ice using a $^{57}$Co source (Eldorado irradiator, A.E.C. Ltd, Canada). Amounts of irradiated cells corresponding to 4.5 nCi of [$^3$H] thymidine-labelled DNA were added as internal standards to $2.5 \times 10^5$ drug-treated [$^{14}$C]-labelled cells. After 2 washings in ice-cold PBS, cells were layered onto a polyvinyl filter (pore size, 0.5 µM, Gelman Sciences, Ann Harbor, Michigan) and lysed with a solution of 2% SDS, 10 mM disodium EDTA ( pH = 10) and 0.5 mg $ml^{-1}$ proteinase K. DNA was eluted from the filter with 2% tetrapropylammonium hydroxide (Fluka, Mulhouse, France), 20 mM disodium EDTA and 0.1% SDS, pH 12.1, at a flow rate of 0.2 ml $min^{-1}$, with a fractional interval of 5 min. Quantification of deglyco-BLM-$A_2$-induced DNA breaks was performed by measuring the fraction of [$^{14}$C] DNA remaining on the filter when 75% of the [$^3$H]-labelled internal standard DNA was still retained. Irradiated [$^{14}$C]-labelled cells were used to establish the calibration curve giving the relationship between the effect of radiation and the frequency of deglyco-BLM-$A_2$-induced DNA breaks (radiation-equivalent DNA damage). This curve was obtained by plotting radiation dose (rads) versus [$^{14}$C]DNA retention at 75% retention of the [$^3$H]DNA internal standard.

### Conventional DNA electrophoresis

Cell DNA fragmentation was monitored by a gel electrophoresis method adapted from Smith et al (1989). Briefly, after cell treatment, samples of $10^6$ cells were incubated at 50˚C for 1 h in 20 µl of 10 mM EDTA, 50 mM Tris-HCl (pH 8.0) containing 0.5% (w/v) sodium lauryl sarkosinate and 0.5 mg $ml^{-1}$ proteinase K. Then, 10 µl of 0.5 mg $ml^{-1}$ DNase-free RNase was added to each sample and incubation at 50˚C continued for 1 h. Samples were heated to 70˚C, and 10 µl of 30% (w/v) glycerol, 0.25% (w/v) bromophenol blue and 0.25% (w/v) xylene cyanol were added to each sample before its loading into a dry well of a 1.5% (w/v) agarose gel containing 0.1 µg $ml^{-1}$ ethidium bromide. Electrophoresis was carried out at 5 V $cm^{-1}$ in 2 mM EDTA, 89 mM Tris-borate (pH 8.0) until the marker dye migrated at least 3–4 cm. Analysis of the optical density of the bands was performed on negatives of the gels, using a video camera and the BioProfil program from Vilber Lourmat, France, for data analysis.

### Pulsed field DNA electrophoresis

DC-3F cells were grown for 42 hours in the presence of [$^3$H]-thymidine (0.01 µCi $ml^{-1}$) (Dupont de Nemours, Les Ulis, France) in the usual culture medium. Labelled cells were electropermeabilized as described above in the presence of various concentrations of BLM or deglyco-BLM. After 5 min at 37˚C, cells were washed three times at 4˚C in PBS without $Ca^{++}$ and $Mg^{++}$ and the final pellet was suspended in 250 µl of NaCl (0.9%).

Radiolabelled DC-3F cells were also exposed to various control irradiation doses (10, 20 and 30 Gy) at 4˚C using an experimental irradiator IBL 6000 ($^{137}$Cs) (Cea, Saclay, France) yielding 1.414 Gy $min^{-1}$. The culture flasks were pre-cooled for 30 min and kept on ice throughout the irradiation period. Immediately after

© 2001 Cancer Research Campaign irradiation, flasks were transferred to 37˚C for 5 min (like the electropermeabilized cells). Medium was then aspirated and replaced with ice-cold PBS to stop DSB rejoining. Cell monolayers were trypsinized on ice with a HEPES-buffered solution (pH = 7.8) containing 0.25% trypsin and 0.04% EDTA. Cells were suspended in cold growth medium containing 20% calf serum, centrifuged and washed 3 times in ice-cold PBS before passing through a small bore pipette to break up clumps.

Experimental and control cell suspensions were mixed with an equal volume of 1% low melting temperature agarose (Bethesda Res Lab) kept at 40˚C to obtain a final concentration of $2 \times 10^6$ cells $ml^{-1}$. Mixtures were immediately transferred to plug moulds kept at 4˚C and were allowed to solidify for 1 hour. The solidified plugs (7 mm × 4 mm × 1 mm) were then taken out from the moulds, placed at 4˚C for 1 h in a lysis solution (EDTA 0.5 M, 2% Sarkosyl, 1 mg $ml^{-1}$ proteinase K) to allow permeation of the lysis agents, and incubated for 38 h at 50˚C. After lysis, plugs were washed twice with TE for 30 min (10 mM Tris, 1 mM EDTA, pH = 8) at 4˚C, incubated for 1 h with 0.1 mg $ml^{-1}$ RNAse, washed twice with TE and placed in 0.2 M EDTA at 4˚C.

For pulsed-field gel electrophoresis, agarose gels (0.7% Seakem, CTG, FMC, Rockland, ME, USA) were cast in 0.5 X TBE (45 mM Tris Base, 45 mM boric acid, 1 mM EDTA). Agarose plugs containing electropermeabilized, irradiated, or control cells, as well as molecular size markers (*Schizosaccharomyces pombe*, Bio-Rad, Ivry sur Seine, France) were placed into the wells. Electrophoresis was then carried out in a PFGE CHEF DRII apparatus (Bio-Rad) at 14˚C in 0.5 X TBE according to Foray et al (1996).

After electrophoresis, gels were stained in 0.1 mg $ml^{-1}$ ethidium bromide for 20 min and destained for 30 min in 0.5 × TBE at 4˚C. Following photography under UV light, lanes were separated from wells for each sample and loaded into separate scintillation vials. 0.5 ml HCl 1 N were added to prevent rejellying. Agarose was melted by heating, mixed with 7 ml of ReadyGel (Beckman) and counted in a liquid scintillation counter. The fraction of activity released (FAR) into the lane was determined according to the following formula:

$$\text{Fraction of activity released} = 100 \times \frac{cpm_{lane}}{cpm_{lane} + cpm_{well}} \quad (1)$$

### Nuclei preparation

Cells were washed twice with cold PBS and incubated for 5 min at 4˚C with 1 ml of lysis solution (20 mM Tris-HCl; 1 mM EDTA; 10 µM pepstatin; 10 µM leupeptin; 1 mM alpha-dithiothreitol; 0.4 mM phenyl-methyl-sulfonyl fluoride). Cells were then collected with a rubber policeman and plates were rinsed with another ml of lysis solution. After 25 min of incubation at 4˚C, cells were homogenized using a Potter dounce A (30 strokes). Nuclei were centrifuged (500 ***g***, 15 min, 4˚C) and suspended in 0.5 mM $Ca^{2+}$-supplemented serum-free S-MEM at a density of $2.2 \times 10^7$ nuclei $ml^{-1}$. After nuclei incubation with Fe-BLM-$A_2$ or Fe-deglyco-BLM-$A_2$ for various times, DNA fragmentation was monitored as described above for cells.

### RESULTS

### Cytotoxicity determinations

Deglyco-BLM-$A_2$ displayed only a very low toxicity to intact, non-electropermeabilized cells exposed for 1 hour to this product. In fact, reduction to 50% of the cloning efficiency ($EC_{50}$) was never reached, even at the highest dose available (Figure 1A). These results indicate that under the same experimental conditions (Figure 1A), deglyco-BLM-$A_2$ is more than 100 times less toxic than BLM-$A_2$.

Cell EPN increases the toxicity of both BLM-$A_2$ and deglyco-BLM-$A_2$ (Figure 1B). Nevertheless, deglyco-BLM-$A_2$ is still about 100 times less toxic than BLM-$A_2$ (Figure 1B). When electropermeabilized cells were exposed to cytotoxic concentrations of deglyco-BLM-$A_2$, no morphological signs of mitotic cell death (Tounekti et al, 1993) were observed: in particular, no increase in cell size or in nucleus size was detected before the cells became trypan blue positive. On the contrary, cell shrinkage, blebbing at the cell surface and condensation of chromatin were all observed under light microscopy (data not shown). In other words, cells treated with deglyco-BLM-$A_2$ displayed the morphological characteristics of apoptosis.

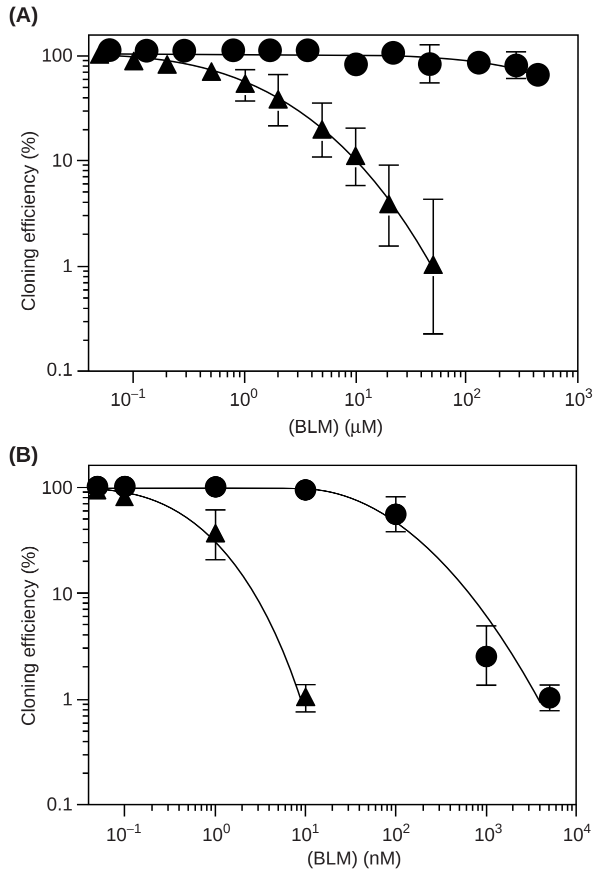

**Figure 1** Determination of BLM-$A_2$ and deglyco-BLM-$A_2$ cytotoxicities on intact cells (**A**) and on electropermeabilized cells (**B**). (▲BLM-$A_2$; (●) deglyco-BLM-$A_2$. Each point is the mean of at least 3 independent determinations. Error bars correspond to standard deviations

### Induction of apoptosis by deglyco-BLM-$A_2$

Oligonucleosomal DNA ladder generation by deglyco-BLM-$A_2$ in electropermeabilized cells was investigated (Figure 2). It is known

*© 2001 Cancer Research Campaign*

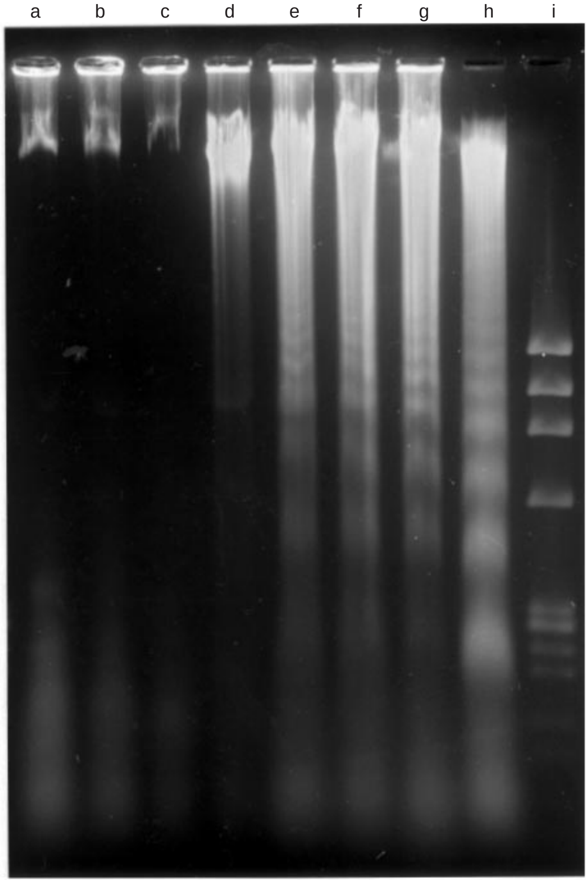

**Figure 2** Time course of deglyco-BLM-$A_2$-induced DNA fragmentation in DC-3F cells. Lane a, cells electropermeabilized in the absence of drug; lanes b–g, DNA samples prepared 12 h (lanes b, c), 24 h (lanes d, e) or 48 h (lanes f, g) after the electropermeabilization of the cells in the presence of 10 µM deglyco-BLM-$A_2$ (lanes b, d, f) or 100 µM deglyco-BLM-$A_2$ (lanes c, e, g); lane h, DNA sample prepared 5 min after cell EPN in the presence of 10 µM BLM-$A_2$; lane i, size marker (Phi X 174 DNA digested by *HaeIII* restriction endonuclease)

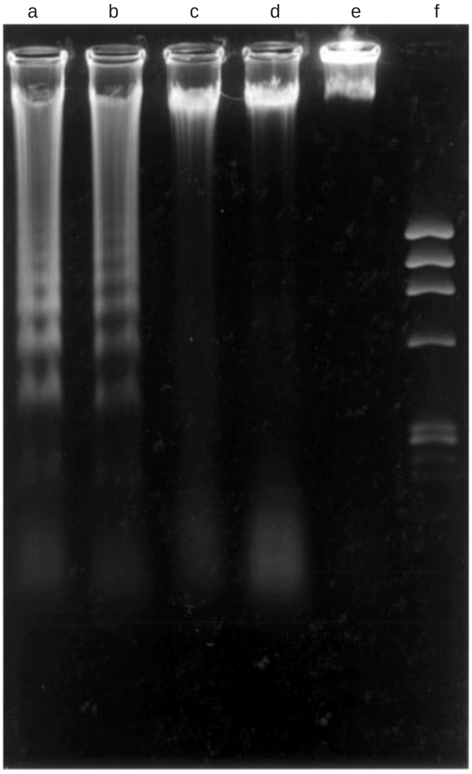

**Figure 3** Inhibition of deglyco-BLM-$A_2$-induced DNA fragmentation in DC-3F cells. Lane a, DNA samples prepared 24 h after cell EPN in the presence of 100 µM deglyco-BLM-$A_2$; lanes b-d, DNA samples prepared 24 h after cell EPN in the presence of 100 µM deglyco-BLM-$A_2$ followed by the addition, 1 h after the electric pulses delivery, of (b) 1.5 mM $CoCl_2$ or (c) 1.5 mM $ZnCl_2$ or (d) 50 µM aurintricarboxylic acid; lane e, cells electropermeabilized in the absence of deglyco-BLM-$A_2$; lane f, size marker (Phi X 174 DNA digested by *HaeIII* restriction endonuclease)

that the commercially available BLM generates DSB immediately after its internalization by electropermeabilized cells, and that these DSB mainly occur in the DNA linker between two consecutive nucleosomes, resulting in the very rapid appearance of an oligonucleosomal DNA ladder (Tounekti et al, 1993, 1995). The same result was found with purified BLM-$A_2$: the DNA ladder was detectable even when incubation was stopped 5 min after delivery of electric pulses, i.e. 5 min after the entrance of the drug inside the cells (Figure 2, lane h). On the contrary, DNA fragmentation was detected neither at 5 min nor at 1 h, nor even at 12 h after deglyco-BLM-$A_2$ treatment (Figure 2, lane b and c). Nevertheless, the oligonucleosomal ladder was detectable 24 h after deglyco-BLM-$A_2$ internalization by cells exposed to 10 or 100 µM external concentrations (Figure 2, lane d and e). Extensive DNA fragmentation was detected 48 h after treatment with either of these 2 concentrations (Figure 2, lane f and g). In addition, a DNA ladder was also detected 72 h after cell EPN in the presence of lower deglyco-BLM-$A_2$ concentrations (1 µM, data not shown).

In additional experiments, DC-3F cells electropermeabilized in the presence of deglyco-BLM-$A_2$ were treated with known inhibitors of the usual endonucleases involved in the apoptotic processes (Peitsch et al, 1994). DNA fragmentation into oligonucleosomal fragments was not inhibited by either the presence of

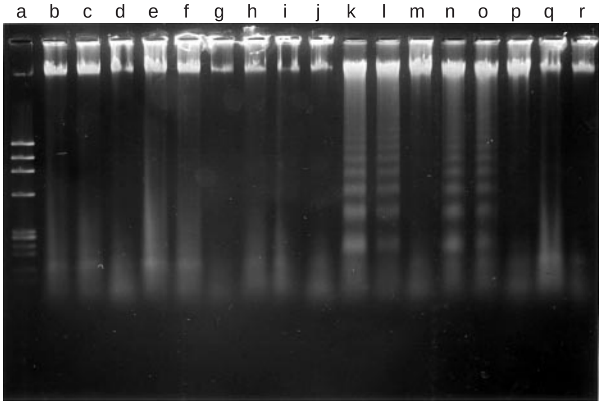

**Figure 4** Analysis of DNA from DC-3F cells nuclei treated with BLM-$A_2$ and deglyco-BLM-$A_2$. Lane a, size marker (Phi X 174 DNA digested by *HaeIII* restriction endonuclease); lanes b–d, nuclei treated with 1 mM deglyco-BLM-$A_2$ for: (b) 2 h, (c) 1 h, (d) 5 min; lanes e–g, nuclei treated with 100 µM deglyco-BLM-$A_2$ for: (e) 2 h, (f) 1 h, (g) 5 min; lanes h–j, nuclei treated with 10 µM deglyco-BLM-$A_2$ for: (h) 2 h, (i) 1 h, (j) 5 min; lanes k–m, nuclei treated with 100 µM BLM-$A_2$ for: (k) 2 h, (l) 15 min, (m) 5 min; lanes n–p, nuclei treated with 10 µM BLM-$A_2$ for: (n) 2 h, (o) 15 min, (p) 5 min; lane q, nuclei incubated with 1 mM $FeCl_2$ for 2 h; lane r, untreated nuclei

© 2001 Cancer Research Campaign

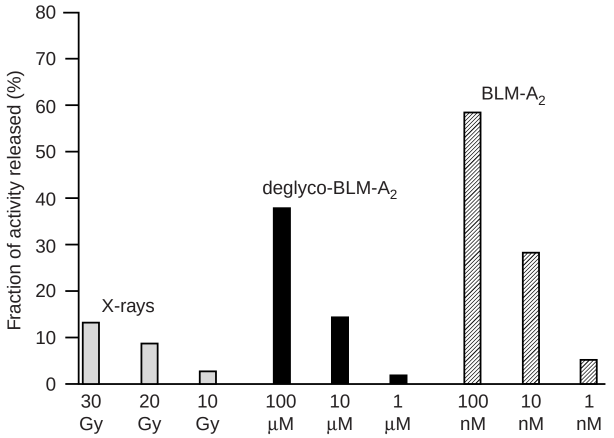

**Figure 5** Induction of DSB, determined by the fraction of activity released after pulsed field gel electrophoresis, in cells exposed to radiation at 4˚C or electropermeabilized in the presence of various concentrations of BLM-$A_2$ or deglyco-BLM-$A_2$

EDTA or EGTA (1–5 mM) or of diethylpyrocarbonate (1 nM–10 mM) (data not shown). However, addition of 50 µM aurintricarboxylic acid (Figure 3, lane d) or of 1.5 mM $ZnCl_2$ (Figure 3, lane c) one hour after the EPN in the presence of deglyco-BLM-$A_2$ resulted in the inhibition of DNA fragmentation. On the contrary, under the same experimental conditions used for $ZnCl_2$, $CoCl_2$, a potent inhibitor of BLM endonucleasic activity, was unable to inhibit the deglyco-BLM-$A_2$-induced DNA fragmentation (Figure 3, lane b). These results indicate that deglyco-BLM-$A_2$ does not directly generate the oligonucleosomal ladder: the ladder could result in fact from the activation of specific cellular endonucleases. Therefore, deglyco-BLM-$A_2$ seems to induce a true apoptotic process.

### Effects of deglyco-BLM-$A_2$ on isolated nuclei

DNA fragmentation was also analysed in nuclei isolated from DC-3F cells. A 15 min treatment of isolated nuclei with BLM-$A_2$ was sufficient for an oligonucleosomal ladder to appear (Figure 4, lanes k–p), as expected for this apoptosis-mimetic drug (Tounekti et al, 1993). This DNA fragmentation was as intense with 10 µM as with 100 µM, in agreement with previous studies performed with the commercial mixture of bleomycins (the similarity of the results obtained with these 2 concentrations, 10 µM, and 100 µM, was already discussed in Tounekti et al, 1993). In contrast, with deglyco-BLM-$A_2$, no oligonucleosomal ladder could be detected during the first 2 hours of treatment, even at the highest concentration tested (1 mM) (Figure 4), nor even after longer periods (data not shown). We conclude that the oligonucleosomal ladder observed after deglyco-BLM-$A_2$ treatment of electropermeabilized cells most likely results from a cellular reaction triggered by the DNA damages produced by the deglyco-BLM-$A_2$ i.e. from a true apoptosis requiring cytoplasmic factors. Indeed, the results from isolated nuclei clearly show that deglyco-BLM-$A_2$ is unable to directly generate oligonucleosomal DNA fragmentation.

### DNA breaks generated in living cells by BLM-$A_2$ or deglyco-BLM-$A_2$

DC-3F cells electropermeabilized in the presence of various external concentrations of deglyco-BLM-$A_2$ showed a concentration-dependent accumulation of DNA breaks: 175, 744, 1031 and 1692 rad-equivalents for respectively 0, 0.1, 1 and 10 µM in comparison with control DC-3F cells not submitted to the electric pulses. These results demonstrate that, once inside the cells, deglyco-BLM-$A_2$ actually generates DNA breaks.

DNA of cells electropermeabilized in the presence of various concentrations of BLM-$A_2$ or of deglyco-BLM-$A_2$ was analysed by pulsed field gel electrophoresis. Control DC-3F cells exposed to 10, 20 and 30 Gy showed a linear dose-dependent increase of the fraction of activity released (FAR) (Figure 5). In the presence of BLM concentrations as low as $10^{-9}$ M, a significant FAR (approximately equivalent to an exposure to 15 Gy) could already be detected (Figure 5). At $10^{-8}$ M it reached 30%, a much higher percentage than that generated by exposure to 30 Gy. In the presence of $10^{-6}$ M deglyco-BLM-$A_2$, a small FAR (approximately equivalent to an exposure to 5 Gy) was detected. With 10 times more deglyco-BLM-$A_2$, FAR was slightly higher than that generated by exposure to 30 Gy, and with $10^{-4}$ M deglyco-BLM it was slightly more important than with $10^{-8}$ M BLM-$A_2$. Thus, the dose-dependent increase in FAR with BLM-$A_2$ is steeper and can be detected at lower concentrations than the FAR increase observed in the presence of deglyco-BLM-$A_2$.

### DISCUSSION

### Deglyco-BLM-$A_2$ is less toxic than BLM-$A_2$

In spite of minor differences in their physicochemical properties (Kenani et al, 1988a), deglyco-BLM-$A_2$ and BLM-$A_2$ share the same mechanism of nucleophilic attack at the same DNA sequences (Bailly et al, 1995). However, on intact cells, deglyco-BLM-$A_2$ is much less toxic than BLM-$A_2$ Deglyco-BLM-$A_2$ cytotoxicity was also determined in transiently and reversibly electropermeabilized cells, in which the plasma membrane does not restrict hydrophilic molecule cytotoxicity. Under these conditions, deglyco-BLM-$A_2$ is still much less toxic than BLM-$A_2$ (Figure 1). Therefore, removal of the glycanic moiety from the bleomycin molecule appears to have important biological consequences.

### Cell death pathways of cells treated with BLM-$A_2$ or deglyco-BLM-$A_2$

Our results suggest that in the presence of deglyco-BLM-$A_2$, electropermeabilized cells follow a different cell death pathway than with standard BLM.

Indeed, a significant FAR is detected at the lowest toxic BLM concentrations, and as we previously showed (Tounekti et al, 1993), cells treated at these low BLM concentrations are killed through a mitotic cell death pathway resulting from the generation of a few unrepaired DSB. We had also demonstrated that BLM is an apoptosis-mimetic drug (Tounekti et al, 1993): at high intracellular concentrations, BLM itself produces a large number of DSB, mimics the endonucleases usually involved in classical apoptosis, and leads to the generation of morphological characteristics of apoptotic cells (Tounekti et al, 1995). Both cell death pathways caused by BLM rely on the ability of BLM to generate DSB.

In addition to causing morphological changes characteristic of apoptosis, deglyco-BLM-$A_2$ can also induce the appearance of an oligonucleosomal ladder (Figure 2). However, in contrast to BLM or to BLM-$A_2$, the ladder can only be detected at least 24 hours after deglyco-BLM-$A_2$ internalization and, moreover, its

 *© 2001 Cancer Research Campaign*

generation is inhibited by the addition of aurintricarboxylic acid or of zinc ions. These results suggest that a NUC-18-like endonuclease might be involved in the deglyco-BLM-$A_2$-induced DNA fragmentation (Peitsch et al, 1994). Moreover, the oligonucleosomal ladder could not be detected when isolated nuclei were exposed to deglyco-BLM-$A_2$ (Figure 4). The necessity for a cytoplasmic factor (like the apoptosis-related endonuclease itself, or a kinase, protease or phosphatase able to activate an endonuclease already present in the nucleus) strongly argues in favour of the induction of an apoptotic process (White, 1996). Our observations allow us to conclude that cells treated with deglyco-BLM-$A_2$ die through a true apoptotic process.

### DNA fragmentation generated by deglyco-BLM-$A_2$

Possible variations in the endonucleasic activities of deglyco-BLM and BLM were investigated as a potential explanation for their differences in toxicity. Indeed, BLM cytotoxicity is related to the generation of DSB (Tounekti et al, 1993, 1995) in spite of the fact that BLM produces 6 SSB per DSB. Moreover, the link between BLM lethal effects and the DSB generated by BLM at low concentrations was confirmed in the results here reported. Indeed, analysis of DNA fragmentation using pulsed field electrophoresis (Figure 5) showed that at the lowest lethal BLM concentration ($10^{-9}$ M), there was already a large FAR even as soon as 5 min after drug delivery, in keeping with the direct endonucleasic activity of BLM, and as demonstrated in previous studies (Tounekti et al, 1993).

Analysis of cell DNA recovered by alkaline elution revealed that large quantities of DNA breaks occur in living cells within minutes of internalization of 0.1 μM deglyco-BLM-$A_2$ (a concentration toxic for around 60% of the electropermeabilized cells). This result confirms the hypothesis that DNA is the actual target of deglyco-BLM-$A_2$. It is very different, however, from the results obtained using pulsed field electrophoresis: indeed, significant amounts of FAR were detected only at much higher external concentrations of deglyco-BLM-$A_2$, concentrations 10 to 100 times higher than those already lethal. Thus, the DNA fragmentation revealed by the FAR cannot be directly responsible for the lethal effects of the lowest toxic deglyco-BLM-$A_2$ concentrations. In other words, the DSB revealed by the FAR 5 minutes after treatment should not be the cause of deglyco-BLM-$A_2$ toxicity. Moreover, no direct DSB were visualized in nuclei treated with deglyco-BLM-$A_2$, i.e. under conditions where an excess of deglyco-BLM-$A_2$ has free access to its target for extended periods of time (Figure 4). Thus, the DNA breaks generated by deglyco-BLM-$A_2$ at its lowest toxic concentrations (those shown by the alkaline elution analysis) should essentially be SSB.

### Quantitative conclusions

In this respect, it seems relevant to calculate the number of SSB potentially generated in the electropermeabilized cells. To perform these calculations, it is assumed that:

(i) The absence of the glycanic part has no incidence on the rate of internalization with respect to BLM. Were there to be a modification, however, it would most likely be an increase in internalization since the reduction in molecular weight due to the absence of the glycanic moiety should accelerate crossing of the electropermeabilized plasma membrane; in this case, the resulting numbers would be minimal evaluations.

(ii) In vivo, as in vitro, each BLM molecule produces 8 to 10 strand breaks (Povirk et al, 1989).

(iii) Within the cell, the total nucleasic activity of deglyco-BLM-$A_2$ is roughly half of that of BLM-$A_2$, as it is in vitro (Oppenheimer et al, 1982; Kenani et al, 1988a).

Under these assumptions and according to Poddevin et al (1991), an external concentration of $10^{-4}$ M deglyco-BLM should lead to the internalization of an average of $3 \times 10^7$ molecules of deglyco-BLM per DC-3F cell and should generate about $5 \times 3 \times 10^7 = 1.5 \times 10^8$ SSB. Since a mammalian cell with $3 \times 10^9$ bp has approximately $1.5 \times 10^7$ nucleosomes, then it is reasonable to consider that on average at least 10 SSB are made per nucleosome, i.e. on the more accessible 20 to 30 bp of the linker region. This approximation would be valid if all the nucleosomal linker regions had the same accessibility; this is, however, not the case. Thus on the short stretches of DNA where deglyco-BLM-$A_2$ makes SSB, the generation of 2 SSB at adjacent positions on the opposite DNA strands is more than probable. These SSB coincidences can lead to the generation of functional DSB that can explain FAR generation in pulsed field electrophoresis. Indeed, it is reminded that DNA fragmentation by deglyco-BLM-$A_2$ was detected at external concentrations as low as $10^{-7}$ M when analysed by alkaline elution (detection of SSB and DSB), whereas FAR was only detectable at $10^{-6}$ M and essentially at $10^{-5}$ M external concentrations when using pulsed field electrophoresis (detection of DSB only). Thus, (i) the results of these calculations strongly argue in favour of our hypothesis that deglyco-BLM-$A_2$ essentially generates SSB, and (ii) the functional DSB occurring from SSB coincidences (when very large numbers of SSB are generated) do not explain the lethal effects of the deglyco-BLM-$A_2$.

Moreover, according to agreed estimations (Goodhead, 1989) that 1 Gy (Cs137 γ rays) results in 39 DSB and 1000 SSB per cell, the $1.5 \times 10^8$ SSB per cell generated at $10^{-4}$M deglyco–BLM–$A_2$ correspond to the number of SSB generated by an irradiation of $1.5 \times 10^5$ Gy. According to Goodhead (1989), such an irradiation is above the minimum required dose ($10^5$ Gy) for which coincidence of single hits (i.e. coincidence of SSB) could actually result in DSB generation. Therefore, this parallelism with the situation described in the case of ionizing radiations reinforces our hypothesis that deglyco-BLM-$A_2$ generates almost exclusively SSB unless the number of deglyco-BLM molecules is so high that DSB are created by SSB coincidences.

### Intrinsic cytotoxicity of SSB and DSB

Taking into account that deglyco-BLM-$A_2$ and BLM-$A_2$ generate SSB and DSB by the same chemical reaction on DNA (Bailly et al, 1995), it seems interesting to compare the intrinsic toxic capacities of SSB and DSB. At their lowest cytotoxic concentrations in electropermeabilized cells, BLM ($10^{-9}$ M) generates 500 DSB (Tounekti et al, 1993) and, deduced from the calculations reported here, deglyco-BLM-$A_2$ ($10^{-7}$ M) should generate 150 000 SSB. Thus, DSB are intrinsically 300 times more cytotoxic than SSB. This difference can be due either to the various efficiencies with which SSB or DSB are repaired, or to the different cell death pathways resulting from the presence of SSB or DSB in the cell genome.

We have concluded that deglyco-BLM-$A_2$ molecules can generate essentially only SSB. Indeed, in theory, it cannot be excluded that

*© 2001 Cancer Research Campaign*

**Table 1** Relationship between the type of cell death and the absolute and relative numbers of SSB and DSB in Chinese hamster fibroblasts

| Drug activity | | DNA fragmentation | | | Potential types of cell death | | | Result |
|---|---|---|---|---|---|---|---|---|
| Directly generated DSB | Directly generated SSB | Immediate oligonucleosomal fragmentation | Delayed oligonucleosomal fragmentation | FAR | Fast pseudoapoptosis | Slow apoptosis | Very slow mitotic cell death | Observed type of cell death |
| > 50 000 | <150 000 | + | NA | + | + | – | NA | Pseudoapoptosis |
| > 50 000 | >150 000 | + | NA | + | + | + | NA | Pseudoapoptosis |
| 500 > > 50 000 | <150 000 | – | – | – | – | – | + | Mitotic cell death |
| 500 > > 50 000 | >150 000 | – | + | –/+(*) | – | + | + | Apoptosis |
| < 500 | <150 000 | – | – | – | – | – | – | Survival (repair) |
| < 500 | >150 000 | – | + | –/+(*) | – | + | – | Apoptosis |

(*)for very large amounts of SSB (coincidences). The threshold values for the number of SSB and DSB are valid for DC-3F cells, a Chinese Hamster cell line, on which all experiments were performed using a large range of concentrations of bleomycin and of deglyco-bleomycin. Absolute values for other cells may be different, depending, e.g., on their own DNA repair potential and on the status of their apoptotic pathways. The numbers in the table are themselves averaged approximations (for example, as reported in Poddevin et al. 1991, 500 DSB roughly corresponds to the number of internalized BLM molecules (300) resulting in 50% of loss of viability; however, the first DC-3F cells can be killed with an average of 100 BLM molecules per cell, and all the DC-3F cells are killed with an average of 2000 molecules per cell).

deglyco-BLM-$A_2$ molecules also generate a very small number of DSB. In fact, with respect to the number of SSB generated, a minimal value for the ratio of SSB to DSB can be estimated in the following way. At 1 µM, deglyco-BLM-$A_2$ generates 1 500 000 SSB. At this concentration of deglyco-BLM-$A_2$, the FAR is lower than that obtained with 1 nM BLM, that corresponds to less than 500 DSB (Tounekti et al, 1993). Thus, for deglyco-BLM-$A_2$, the ratio of SSB to DSB should be greater than 1 500 000/500, i.e. greater than 3000. Since each individual molecule of deglyco-BLM-$A_2$ generates only 5 SSB, the probability that each molecule of deglyco-BLM-$A_2$ generates a DSB is lower than 5/3000 = 0.0017. This value confirms the notion that deglyco-BLM-$A_2$ must likely generate essentially SSB.

### SSB induce apoptosis

Since our previous results using bleomycin showed that DSB in limited numbers do not induce apoptosis (Tounekti et al, 1993, 1995), and since deglyco-BLM-$A_2$ molecules generate essentially SSB, and furthermore, since cells treated with deglyco-BLM-$A_2$ die through a true apoptotic process, it can be concluded that SSB induce apoptosis.

Until now, the role of SSB in apoptosis has been under debate. There is a possibility that SSB precede the appearance of DSB in the internucleosomal linker region (Tomei, 1991). However, SSB could act as signals to induce apoptosis (Yoshida et al, 1993). Indeed, SSB generated in camptothecin-treated HL-60 cells induced internucleosomal DNA cleavage, in spite of the fact that these SSB were rapidly repaired after drug removal (Yoshida et al, 1993). However, in those experiments, the existence of another signal could not be excluded because (i) camptothecin does not interact directly with DNA but with topoisomerase I, the DNA-binding protein actually responsible for SSB, and (ii) topoisomerase I could have more than one role in cell physiology. Our results strongly sustain the mechanism proposed by Yoshida et al, since we show that the SSB directly generated in the DNA by deglycoBLM-$A_2$ molecules are in fact signals leading to death through apoptosis.

### SSB, DSB and cell death

In summary, under experimental conditions in which SSB and DSB result from the same mechanism of DNA attack, one of three different types of cell death can result, depending on the number and kind of DNA breaks (Table 1). DSB are intrinsically very toxic in themselves but are unable to induce apoptosis; SSB are much less toxic and are in fact not directly toxic in themselves but through induction of apoptosis. The difference in the intrinsic cytotoxicities of SSB and DSB explains why BLM toxicity is actually related to the generation of DSB, even if BLM causes an average of 6 SSB for one DSB (Grimwade and Beerman, 1986; Cullinan et al, 1991). On the contrary, it may be suggested that molecules that produce more than 500 SSB for one DSB could induce apoptosis.

### ACKNOWLEDGEMENTS

The authors thank B Leon for her excellent technical assistance and L Perricaudet for linguistic revision of the manuscript. This work was supported by the Centre National de la Recherche Scientifique and the Institut Gustave-Roussy, and by a grant from the Association pour la Recherche contre le Cancer. We thank the Société Française du Cancer and the Comités de l'Essone et du Val d'Oise de la Ligue Nationale contre le Cancer for financial support to OT, and the INSERM (France), CNRS (France) and DGRST (Tunisia) for travel support to AK, OT and LMM for scientific exchanges.

### REFERENCES

Bailly C, Kenani A and Waring MJ (1995) Analogue versus digital recognition of DNA by bleomycin: an effect of the carbohydrate moiety. *FEBS Lett* **372**: 144–147

Cohen-Jonathan E, Bernhard EJ and McKenna WG (1999) How does radiation kill the cells? *Curr Opin Chem Biol* **3**: 77–83

Cole A, Shonka F, Corry PM and Cooper WG (1975) CHO cell repair of single strand and double strand breaks induced by gamma and alpha radiations. In: Hanawalt P and Setlon R (eds), *Molecular mechanisms for the repair of DNA, II*, pp 665–676. Plenum Press Ltd: New York

Cole A, Meyn RE, Chen R, Corry PM and Hillelman W (1980) Mechanisms of cell injury. In: Meyn RE, Withers HR (eds), *Radiation Biology in Cancer Research*, pp 33–58. Raven Press Ltd: New York

Cullinan EB, Gawron LS, Rustum YM and Beerman TA (1991) Extrachromosomal chromatin: Novel target for bleomycin cleavage in cells and solid tumors. *Biochemistry* **30**: 3055–3061

Foray N, Fertil B, Alsbeih MGA, Badie C, Chavaudra N, Iliakis G and Malaise EP (1996) Dose-rate effect on radiation-induced DNA double strand breaks in the human fibroblast HF19 cell line. *Int J Radiat Biol* **69**: 241–249

 © 2001 Cancer Research Campaign

Goodhead DT (1989) The initial physical damage produced by ionizing radiations. *Int J Radiat Biol* **56**: 623–634
Grimwade J and Beerman T (1986) Measurement of bleomycin, neocarzinostatin, and auromomycin cleavage of cell-free and intracellular simian virus 40 DNA and chromatin. *Mol Pharmacol* **30**: 358–363
Kenani A, Bailly C, Helbecque N, Catteau JP, Houssin R, Bernier JL and Hénichart JP (1988a) The role of the gulose-mannose part of bleomycin in inactivation of iron-molecular oxygen complex. *Biochem J* **253**: 497–504
Kenani A, Lambling G and Henichart JP (1988b) A convenient method for the cleavage of the D-mannosyl-L-gulose disaccharide from bleomycin $A_2$. *Carbohydr Res* **177**: 81–89
Kolesnick RN, Haimovitz-Friedman A and Fuks Z (1994) The sphingomyelin signal transduction pathway mediates apoptosis for tumor necrosis factor, Fas, and ionizing radiation. *Biochem Cell Biol* **72**: 471–474
Mir LM, Tounekti O and Orlowski S (1996) Bleomycin: revival of an old drug. *Gen Pharmacol* **27**: 745–748
Oppenheimer NJ, Chang C, Chang L-H, Ehrenfeld GM, Rodriguez LO and Hecht SM (1982) Deglyco-bleomycin Degradation of DNA and formation of a structurally unique Fe(II)-Co complex. *J Biol Chem* **257**: 1606–1609
Orlowski S and Mir LM (1993) Cell electropermeabilization: a new tool for biochemical and pharmacological studies. *Biochim Biophys Acta* **1154**: 51–63
Orlowski S, Belehradek Jr J, Paoletti C and Mir LM (1988) Transient electropermeabilization of cells in culture. Increase of the cytotoxicity of anticancer drugs. *Biochem Pharmacol* **37**: 4727–4733
Peitsch MC, Mannhertz HG and Tschopp J (1994) The apoptosis endonucleases: cleaning up after cell death? *Trends Cell Biol* **4**: 37–41
Poddevin B, Orlowski S, Belehradek JrJ and Mir LM (1991) Very high cytotoxicity of bleomycin introduced into the cytosol of cells in culture. *Biochem Pharmacol* **42(S)**: 67–75
Povirk LF, Wübker W, Köhnlein W and Hutchinson F (1977) DNA double-strand breaks and alkali-labile bonds produced by bleomycin. *Nucleic Acids Res* **4**: 3573–3580
Povirk LF, Han YH, and Steighner RJ (1989) Structure of bleomycin-induced DNA double-strand breaks: predominance of blunt ends and single-base 5′ extensions. *Biochemistry* **28**: 5808–5814
Santana P, Pena LA, Haimovitz-Friedman A, Martin S, Green D, McLoughlin M, Cordon-Cardo C, Schuchman EH, Fuks Z and Kolesnick R (1996) Acid sphingomyelinase-deficient human lymphoblasts and mice are defective in radiation-induced apoptosis. *Cell* **86**: 189–199
Smith CA, Williams GT, Kingston R, Jenkinson EJ and Owen JJ (1989) Antibodies to CD3/T-cell receptor complex induce death by apoptosis in immature T cells in thymic cultures. *Nature* **337**: 181–184
Sugiyama H, Kilskukie RE, Chang L-H, Ma LT, Hecht SM, van der Marel GA and van Boom JH (1986) DNA strand scission by bleomycin: catalytic cleavage and strand selectivity. *J Am Chem Soc* **108**: 3852–3854
Tomei LD (1991) Apoptosis: A program for death or survival? In: Tomei LD, Cope FO (eds) *Apoptosis: The molecular basis of cell death*, pp 279–316. Cold Spring Harbor Laboratory Press: New York
Tounekti O, Pron G, Belehradek JrJ and Mir LM (1993) Bleomycin, an apoptosis-mimetic drug that induces two types of cell death depending on the number of molecules internalized. *Cancer Res* **53**: 5462–5469
Tounekti O, Belehradek JrJ and Mir LM (1995) Relationships between DNA fragmentation, chromatin condensation and changes in flow cytometry profiles detected during apoptosis. *Exp Cell Res* **217**: 506–516
White E (1996) Life, death, and the pursuit of apoptosis. *Genes & Dev* **10**: 1–15
Yoshida A, Ueda T, Wano Y and Nakamura T (1993) DNA damage and cell killing by camptothecin and its derivative in human leukemia HL-60 cells. *Jpn J Cancer Res* **84**: 566–573

© 2001 Cancer Research Campaign
